# Body mass index impact on health care utilization among people with diabetes in Jeddah, Saudi Arabia

**DOI:** 10.3389/fpubh.2026.1731286

**Published:** 2026-03-23

**Authors:** Saleh Khateeb, Adhari A. Alselmi, Taif Alharbi, Judy Abbas, Rana Z. Flimban, Maysaa Ageel, Mai A. Khatib, Hala Ehab Khashoggi, Sarah M. Hussein

**Affiliations:** 1 Department of Clinical Sciences, MBBS Program, Fakeeh College for Medical Sciences, Fakeeh Care Group, Jeddah, Saudi Arabia; 2 Department of Family Medicine, Dr. Soliman Fakeeh Medical Center, Fakeeh Care Group, Jeddah, Saudi Arabia; 3 Bachelor of Medicine, Bachelor of Surgery Program, Fakeeh College for Medical Sciences, Fakeeh Care Group, Jeddah, Saudi Arabia; 4 Department of Clinical Nutrition, Faculty of Applied Medical Sciences, King Abdulaziz University, Jeddah, Saudi Arabia; 5 Food, Nutrition, and Lifestyle Unit, King Fahd Medical Research Center, King Abdulaziz University, Jeddah, Saudi Arabia; 6 Obesity and Lifestyle Unit, King Abdulaziz University Hospital, King Abdulaziz University, Jeddah, Saudi Arabia; 7 Saudi Diabetes Research Group, King Fahd Medical Research Center, King Abdulaziz University, Jeddah, Saudi Arabia; 8 King Fahad Hospital, Ministry of Health, Jeddah, Saudi Arabia; 9 Department of Public Health, Community Medicine, Environmental Medicine, and Occupational Medicine, Faculty of Medicine, Suez Canal University, Ismailia, Egypt

**Keywords:** emergency, health service, inpatient, morbidities, obesity, outpatients, overweight

## Abstract

**Background:**

Diabetes mellitus and obesity represent public health challenges, with implications for healthcare utilization. While obesity increases the risk of diabetes and related comorbidities, its impact on healthcare service use among diabetic patients in the Saudi context remains underexplored.

**Objectives:**

Our study aimed to examine the associations between BMI categories and patterns of healthcare utilization among People with diabetes.

**Methods:**

A cross-sectional analysis was conducted in 2024 using medical records from DSFH, Jeddah, Saudi Arabia, including 125,660 People with diabetes. Descriptive statistics summarized demographic, anthropometric, and utilization characteristics. Associations between BMI categories and demographic/utilization variables were assessed using chi-square tests. Multivariable logistic regression models estimated odds ratios (ORs) and 95% confidence intervals (CIs) for healthcare utilization across BMI categories.

**Results:**

The mean age was 58.7 years (SD = 14.7), and the mean BMI was 31.5 kg/m^2^ (SD = 5.9). Overweight and obesity were prevalent in 58.8 and 30.0% of patients, respectively, With significant associations between BMI and demographic/utilization variables (*p* < 0.001). Regression models revealed that overweight [OR = 1.43, 95% CI (1.37, 1.50)] and obese patients [OR = 1.83, 95% CI (1.75, 1.91)] utilized more outpatient services. In contrast, underweight patients had significantly higher odds of inpatient admissions [OR = 2.14, 95% CI (1.84, 2.48)]. Insurance was associated with increased outpatient and emergency utilization.

**Conclusion:**

BMI and demographic factors are significant predictors of healthcare utilization among People with diabetes.

## Introduction

Diabetes Mellitus (DM) is a lifelong morbidity that puts a huge burden on the healthcare system (1). The global prevalence of diabetes is estimated at 425 million in 2017 according to the International Diabetes Federation statistics and is expected to escalate to 629 million in 2045 (2). The prevalence rates of DM are particularly high in Saudi Arabia; 23%–25% in national surveys (3) and it increases up to 31.6% in urban populations (4).

Obesity, body mass index (BMI) ≥ 30, affects approximately one-third of adults worldwide (5). It is recognized as a state of chronic low-grade inflammation that contributes to the development of dyslipidemia, insulin resistance, and hyperglycemia (6). One of the major causes of the massive prevalence of diabetes is obesity with the prevalence rate of obesity in Saudi Arabia is ranging between 24.7% and 38.9% (7). Among diabetic populations, obesity prevalence reaches as high as 85.8% in certain regions (8). Central obesity, often measured by waist circumference, demonstrates stronger associations with diabetes than BMI alone (9).

The obesity epidemic has many harmful consequences other than type 2 diabetes including cardiovascular disease, hypertension, dyslipidemia, stroke, sleep apnea, osteoarthritis, and depression. Interestingly, Given the concerning trend of growing global prevalence of diabetes and obesity (10) with the finding that diabetes patients with higher BMI, especially obese patients, were more frequently in poor physical health (11).

It is not unexpected that all these conditions increase healthcare utilization. For instance, the impact of obesity on these conditions in Saudi Arabia cost a total of $3. 8 billion in 2022 (10). The rise in healthcare usage and expenses is due to treating medical conditions like type 2 diabetes, which makes up 90% of all diabetes cases worldwide, and it is caused by excess weight. However, there are also additional costs from treating obesity directly. This includes surgeries and medications for weight loss (12).

Therefore, with both diabetes and obesity being among chronic conditions that have direct impact on patients’ quality of life with decreased work productivity, this increases healthcare and all socioeconomic costs (13). As regards, Healthcare utilization among patients with diabetes, variability is usually encountered across different BMI categories. Obese patients often require greater outpatient services, whereas underweight patients may be disproportionately represented in hospital admissions and emergency department (ED) visits, reflecting complex care needs. Moreover, BMI itself predicts adherence, as individuals with normal BMI are more likely to follow dietary and exercise regimens (14).

Despite these insights, important gaps remain. Regional variations in healthcare utilization among diabetics across Saudi Arabia highlight the need for more granular analyses (15). Few studies have comprehensively examined the intersection of BMI, demographics, and healthcare utilization in Middle Eastern populations, limiting the evidence base for targeted policy interventions (16, 17). Addressing this gap is critical, as Saudi Arabia faces escalating public health and economic burdens from diabetes, obesity, and related comorbidities. Understanding how demographic and anthropometric factors shape healthcare use can inform strategies to optimize service delivery, reduce hospital strain, and guide preventive health policies.

## Materials and methods

### Study design, setting, and population

This study is a cross-sectional study that was conducted in 2024 utilizing data from Dr. Soliman Fakeeh Hospital Medical Records for patients who visited DSFH at Jeddah, Saudi Arabia between 1st of January, 2022 to 31st of December. A comprehensive sample of eligible patients in the assigned duration was applied.

### Study variables

*Study population*: The study population included 125,660 diabetes mellitus patients visited DSFH at Jeddah, Saudi Arabia during the study period.

### Inclusion criteria

The data included all patients who had a visit to any healthcare services (Out-patient Department (OPD), In-patient admission (IP), and Emergency Department (ED) with a primary diagnosis of diabetes mellitus including type 1 and type 2 as coded with ICD-10.

### Exclusion criteria

Any visit with an age of less than 18 years old and patients with gestational diabetes. Also, any patient with missing weight or height that led to missing BMI were excluded.

### Sampling

A comprehensive sample of eligible patients in the assigned duration was adopted.

### Study variables

The dependent variable was healthcare utilization, operationalized as type of service used (OPD, IP, and ED). Independent variables included:

- Body Mass Index (BMI), categorized as underweight, normal weight, overweight, and obese.
- Demographic variables: age group and gender.
- Socioeconomic variables: insurance/payment type and smoking status.

### Data management and analysis

Data were imported into Stata version 16 (StataCorp, College Station, TX, United States) for statistical analysis. Descriptive statistics summarized demographic, socioeconomic, BMI, and healthcare utilization variables. Categorical variables were presented as frequencies and percentages, while continuous variables (age and BMI) were expressed as means and standard deviations. Whereas those variables that do not follow the normal distribution were presented as median (range).

Analytical procedures included:

1. *Bivariate analysis*: chi-square tests were applied to examine associations between high BMI categories (Overweight and Obese) and demographic/socioeconomic predictors of healthcare utilization.
2. *Multivariable analysis*: logistic regression models were fitted for each utilization type (OPD, IP, and ED), adjusting for BMI category, age group, gender, insurance status, and smoking. Odds ratios (ORs) with 95% confidence intervals (CIs) were reported.

### Research hypothesis

The primary hypothesis was that overweight and obese adults with diabetes mellitus are more likely to utilize healthcare services than normal-weight adults with diabetes. Sub-hypotheses examined whether this effect varied across OPD, IP, and ED settings.

## Results

### Diabetes patients descriptive characteristics

We obtained completed data from 125,660 medical records of Diabetes Mellitus patients attending DSFH at Jeddah, Saudi Arabia. The mean age of the cohort was 58.7 years (SD = 14.7), and the mean body mass index (BMI) was 31.5 kg/m^2^ (SD = 5.9). Slightly more than half of the patients were male (53.9%), and the overwhelming majority were insured (92.7%). Smokers represented (5.7%) of patients, Outpatient visits constituted the most frequently utilized mode of care (76.9%), followed by inpatient admissions (19.6%) and emergency department (ED) visits (3.5%). With respect to BMI distribution, 58.8% of patients were classified as overweight, 30.0% as obese, 0.7% as underweight, and 0.2% as normal weight (Table 1).

**Table 1:** Distribution of the studied patient’s group regarding their descriptive characteristics, DSFH, Jeddah, KSA, 2022 (*N* = 125,660).

Variable	*n* (%) or M (SD)
Age, years	58.7 (14.7)
18–24	1,575 (1.3%)
25–44	20,879 (16.6%)
45–64	57,299 (45.6%)
65+	45,907 (36.5%)
BMI, kg/m^2^	31.5 (5.9)
Normal	298 (0.2%)
Underweight	818 (0.7%)
Overweight	73,850 (58.8%)
Obese	37,737 (30.0%)
Gender	
Female	57,880 (46.1%)
Male	67,780 (53.9%)
Payment type	
Cash	9,153 (7.3%)
Insurance	116,507 (92.7%)
Visit type	
OPD	96,652 (76.9%)
IP	24,670 (19.6%)
ED	4,338 (3.5%)
Smoker	
No	118,465 (94.3%)
Yes	7,195 (5.7%)

Data are presented as mean (SD) for continuous variables and *n* (%) for categorical variables.

### Bivariate associations

Significant associations were observed between BMI category and all examined demographic and healthcare utilization variables. Specifically, BMI distribution varied significantly by age group [*χ*^2^(12) = 2354.77, *p* < 0.001], visit type [*χ*^2^(8) = 2152.29, *p* < 0.001], payment type [*χ*^2^(4) = 343.15, *p* < 0.001], gender [*χ*^2^(4) = 5532.79, *p* < 0.001], and smoking status [*χ*^2^(4) = 132.40, *p* < 0.001]. These results demonstrate that BMI category is consistently associated with patterns of healthcare utilization and patient characteristics in this population (Table 2).

**Table 2:** Chi-square tests of association between BMI category and demographics/utilization variables, DSFH, Jeddah, KSA, 2022 (*N* = 125,660).

Variable	*χ*^2^ (df)	*p*-value
Age group	2354.8 (12)	< 0.001
Visit type	2152.3 (8)	< 0.001
Payment type	343.2 (4)	< 0.001
Gender	5532.8 (4)	< 0.001
Smoker	132.4 (4)	< 0.001

Pearson Chi-square test used. All associations statistically significant at *p* < 0.001.

### Multivariable logistic regression

Logistic regression models were fitted to examine the independent contribution of BMI and demographic factors to healthcare utilization across inpatient, outpatient, and emergency services (Table 3).

**Table 3:** Logistic regression predicting care access by BMI, Age, Gender, payment type, and smoking, DSFH, Jeddah, KSA, 2023 (*N* = 125,660).

Predictor	OR	95% CI	*p*-value
Inpatient (IP)			
Underweight	2.14	1.84–2.48	< 0.001
Overweight	0.70	0.67–0.73	< 0.001
Obese	0.54	0.51–0.56	< 0.001
Age 25–44	1.28	1.09–1.50	0.002
Age 45–64	1.24	1.07–1.45	0.006
Age 65+	3.54	3.04–4.13	< 0.001
Male	1.36	1.32–1.40	< 0.001
Insurance	0.45	0.43–0.47	< 0.001
Smoker	0.77	0.71–0.82	< 0.001
Outpatient (OPD)			
Underweight	0.47	0.40–0.54	< 0.001
Overweight	1.43	1.37–1.50	< 0.001
Obese	1.83	1.75–1.91	< 0.001
Age 65+	0.42	0.37–0.48	< 0.001
Male	0.78	0.76–0.81	< 0.001
Insurance	1.92	1.84–2.02	< 0.001
Smoker	1.22	1.14–1.30	< 0.001
Emergency (ED)			
Underweight	1.34	0.98–1.83	0.064
Overweight	0.85	0.77–0.94	0.002
Obese	0.80	0.73–0.89	< 0.001
Age 25–44	0.65	0.53–0.79	< 0.001
Age 45–64	0.40	0.33–0.49	< 0.001
Age 65+	0.50	0.41–0.61	< 0.001
Male	0.88	0.82–0.94	< 0.001
Insurance	1.66	1.44–1.91	< 0.001
Smoker	1.13	0.99–1.28	0.063

OR, Odds ratio; CI, Confidence interval. Models adjusted for all variables listed.

For inpatient care, underweight patients had significantly higher odds of admission [OR = 2.14, 95% CI (1.84, 2.48), *p* < 0.001]. In contrast, overweight [OR = 0.70, 95% CI (0.67, 0.73), *p* < 0.001] and obese patients [OR = 0.54, 95% CI (0.51, 0.56), *p* < 0.001] were less likely to require inpatient care. Older age, particularly 65 years and above, was strongly associated with greater inpatient utilization [OR = 3.54, 95% CI (3.04, 4.13), *p* < 0.001]. Similarly, smokers had higher inpatient service utilization [OR = 0.77, 95% CI (0.71–0.82), *p* < 0.001].

In outpatient care, overweight [OR = 1.43, 95% CI (1.37, 1.50), *p* < 0.001] and obese patients [OR = 1.83, 95% CI (1.75, 1.91), *p* < 0.001] were more likely to utilize services, while underweight patients were less likely [OR = 0.47, 95% CI (0.40, 0.54), *p* < 0.001]. Insurance status exerted a notable effect, with insured patients nearly twice as likely to access outpatient services [OR = 1.92, 95% CI (1.84, 2.02), *p* < 0.001]. Smokers were more likely utilized outpatient care [OR = 1.22, 95% CI (1.14, 1.30), *p* < 0.001].

For emergency care, overweight [OR = 0.85, 95% CI (0.77, 0.94), *p* = 0.002] and obese patients [OR = 0.80, 95% CI (0.73, 0.89), *p* < 0.001] exhibited reduced odds of ED visits Older patients were also significantly less likely to utilize ED services. Insurance coverage, however, increased the likelihood of ED use [OR = 1.66, 95% CI (1.44, 1.91), *p* < 0.001].

## Discussion

Obesity is consistently linked to increased healthcare utilization across global and regional studies. Our results revealed that BMI category is consistently associated with patterns of healthcare utilization and patient characteristics in this population. Similar to this study, Systematic reviews done by Hasan indicate that obese children have significantly higher rates of service use, including a 34% increase in emergency department (ED) visits and an 11% increase in outpatient visits compared to their normal-weight peers (18). In the Gulf region, Al Hammadi et al. (19) demonstrated that childhood obesity prevalence reaches 25%–33%, is more common among boys, and tends to rise with age. Within Saudi Arabia, the adult obesity prevalence ranges between 20% and 39%, while adolescent rates have been reported at 19.4% (20). This growing burden translates into an estimated $6.4 billion USD in treatment and management costs. Among hypertensive populations in Saudi Arabia, obesity prevalence ranges from 37.9% to 63.6%, with higher rates among women and strong associations with diabetes and hypercholesterolemia (21). Broader Gulf studies further highlight alarming adult overweight prevalence (25%–50%) and obesity prevalence (13%–50%), with consistent increases documented over time (22, 23).

Insurance coverage, smoking status, and BMI trajectories are additional determinants of healthcare utilization. Evidence from U.S. Populations shows that higher BMI correlates with greater healthcare resource use and costs in both commercially insured groups (24) and Medicare beneficiaries (25, 26). Longitudinal studies confirm that BMI patterns across young and middle-aged adults predict ED visits and hospitalizations over time (27). Smoking status exerts a similar influence: smokers demonstrate greater specialty clinic visits, hospitalizations, and higher healthcare charges (28). Within surgical populations, smoking has been associated with longer lengths of stay and increased risk of reoperation (29). More broadly, modifiable risk factors such as obesity and smoking drive higher healthcare costs within relatively short periods, sometimes as early as 18 months (30). Insurance characteristics also shape utilization patterns, with Medicare patients less likely to access certain procedures, including bariatric surgery, compared to privately insured individuals (31).

Collectively, these findings underscore the multifaceted impact of obesity and related risk factors on healthcare systems. The evidence highlights both the regional burden in the Gulf and Saudi Arabia, as well as the global challenges linked to BMI, smoking, and insurance coverage. Addressing these factors is critical not only for improving patient outcomes but also for reducing long-term economic strain on healthcare systems.

### Study limitation

We recognize that our study is limited to a particular population with special characteristics like geographic location, thus limiting the applicability of the study results to the broader population of Saudi Arabia. Therefore, conducting a larger study on Saudi population from different regions of the KSA as well as diverse social sectors will have more comprehensive insights.

## Conclusion

This study highlights the strong associations between BMI and patterns of healthcare utilization among adults with diabetes. Obesity and overweight were highly prevalent in the population and were significantly associated with increased outpatient service use, whereas underweight status was linked to a higher likelihood of inpatient admissions. In contrast, both overweight and obese patients were less likely to require inpatient and emergency services compared with their normal-weight counterparts. Demographic factors, including age, gender, insurance coverage, and smoking status, further shaped utilization patterns, underscoring the multifactorial nature of healthcare access and demand.

These findings emphasize the importance of tailoring healthcare strategies to account for BMI and demographic characteristics in diabetic populations. Policies that promote preventive care and focus on outpatient care can alleviate the burden of hospitalizations and emergency care, while interventions targeted high risk BMI categories can help to decrease their hospitalization risk Given the high prevalence of obesity and diabetes in Saudi Arabia, integrating these insights into national health policies is critical for reducing long-term healthcare costs and improving patient outcomes.

## Data Availability

The raw data supporting the conclusions of this article will be made available by the authors, without undue reservation.
